# Sex and age differences in attitudes and intention to adopt personalised nutrition in a UK sample

**DOI:** 10.1007/s10389-021-01676-x

**Published:** 2021-12-14

**Authors:** Barbara J. Stewart-Knox, Rui Poínhos, Arnout R. H. Fischer, Mutassam Chaudhrey, Audrey Rankin, Jenny Davison, Brendan P. Bunting, Lynn J. Frewer, Bruno M. P. M. Oliveira

**Affiliations:** 1 Division of Psychology, School of Social Sciences, University of Bradford, Richmond Road, Bradford, BD71DP UK; 2 Faculty of Nutrition and Food Sciences, University of Porto, Rua do Campo Alegre, n.° 823, 4150-180 Porto, Portugal; 3 Social-Sciences, Marketing and Consumer Behaviour, Wageningen University, Hollandseweg 1, 6706 KN Wageningen, The Netherlands; 4 School of Pharmacy, Queen’s University Belfast, 97 Lisburn Road, Belfast, BT9 7BL UK; 5 School of Psychology, Ulster University, Cromore Road, Coleraine, County Londonderry BT52 1SA UK; 6 School of Natural and Environmental Sciences, Newcastle University, Newcastle Upon Tyne, NE191AA UK

**Keywords:** Personalised nutrition, Education, Income, Occupation, Age, Sex

## Abstract

**Aim:**

There has been an increase in the development of technologies that can deliver personalised dietary advice. Devising healthy, sustainable dietary plans will mean taking into consideration extrinsic factors such as individual social circumstances. The aim of this study was to identify societal groups more or less receptive to and likely to engage with personalised nutrition initiatives.

**Sample and methods:**

Volunteers were recruited via a social research agency from within the UK. The resultant sample (*N* = 1061) was 49% female, aged 18-65 years.

**Results:**

MANOVA (Tukey HSD applied) indicated that females and younger people (aged 18-29 years) had more favourable attitudes and were more likely to intend to adopt personalised nutrition. There were no differences in attitude toward or intention to adopt personalised nutrition between different education levels, income brackets or occupational groups.

**Conclusion:**

These results imply that females and younger people may be most likely to adopt personalised nutrition in the future. Initiatives to promote healthy eating should target males and older people.

## Introduction

The goal of personalised nutrition is to maintain current good heath, prevent dietary related health problems, or mitigate existing ones using nutritional and other relevant information about an individual to deliver specific healthy eating guidance and, potentially, nutritional products and services (Ordovas et al. 2018). Individually tailored nutrition advice delivered by personalised targeted messages has been shown to be more effective than generic, non-personalised nutrition advice in increasing healthy eating behaviour (Hoevenaars et al. 2020; Hu et al. 2020; Celis-Morales et al. 2017). At the same time, consumer interest in personalised nutrition has been increasing (Stewart-Knox et al. 2016. Poínhos et al. 2014; Stewart-Knox et al. 2013). This has driven an increase in the development of commercially offered services that can deliver evidence-based, personalised dietary plans that are tailored to individual lifestyle, phenotype, genotype, social circumstances and psychology (Abrahams 2020).

Previous research has pointed to sociodemographic differences in dietary health behaviour. Analysis of the UK National Diet and Nutrition Survey (*N* = 2083) (Roberts et al. 2018) indicated that intake of healthier foods (fish, fruit and vegetables) was more frequent among older people, those with a higher income and of higher socioeconomic status (SES). Surveys on factors related to dietary health promotion have consistently implied that males engage in less healthy eating practices than females (Maugeri et al. 2020; Barrea et al. 2019; Martinez-Lacoba et al. 2018; Hiller et al. 2017; Ashton et al. 2017; van Dillen et al. 2008) and that older people eat healthier diets than those who are younger (Kang et al. 2019; van Dillen et al. 2008). Less healthy dietary habits also tend to be more prevalent among the less educated (Kang et al. 2019; Tan et al. 2017), those with lower incomes (Tan et al. 2017) and those from a more deprived SES background (Maugeri et al. 2020; Martinez-Lacoba et al. 2018). Similarly, attitudes toward personalised nutrition and its future adoption are likely to vary according to sociodemographic characteristics and to follow the same trends. Studies on attitudes and adoption of personalised nutrition in relation to sociodemographic factors, however, are scarce. Surveys conducted in Hungary (*N* = 1000) (Szakaly et al. 2021), (*N* = 500) (Szakaly et al. 2016) and another in six EU countries (*N* = 5967) (Stewart-Knox et al. 2009) both suggested that attitudes toward personalised nutrition were more favourable in women than men. The Hungarian survey (Szakaly et al. 2016) also identified more favourable attitudes among those with a higher education level.

Dietary health promotion must consider the wider socioeconomic implications of associated technologies and potential for indirect negative consequences. Personalised nutrition services are currently (at the time of writing) almost exclusively provided by the commercial sector (Abrahams 2020). For the 30% of consumers who already use digital health devices (Abrahams 2020), adoption of personalised nutrition should be technologically feasible. That socioeconomically disadvantaged people are less likely to have internet broadband at home and to be digitally literate (Azzopardi-Muscat and Sørensen 2019; Weiss and Eikemo 2017) means they may be slower to adopt novel health technologies (Weiss et al. 2018). Food insecurity is also associated with more disadvantaged socioeconomic circumstances (Power et al. 2018). A possible unintended consequence of personal nutrition, therefore, is that it may not reach people living in disadvantaged circumstances (Stewart-Knox et al. 2016). If not made available and accessible to everyone in society, personalised nutrition will do little to address food insecurity and could serve to widen existing health inequalities.

This study considered differences in attitudes and intention toward personalised nutrition between sex, education level, income and occupational grouping as markers of socioeconomic disadvantage. A precondition for any gains is that we understand the degree to which intended recipients are receptive to personalised nutrition. The aim of this analysis was to identify societal groups within our study population who may be more or less receptive to and likely to engage with personalised nutrition initiatives. Given previous research indicating socioeconomic inequalities in healthy eating and access to health-enabling technologies, it is predicted that males, younger people and those with lower education level, with lower income and in manual occupations will hold less favourable attitudes toward personalised nutrition and will be less likely to intend to adopt it in the future.

## Methods

Data on sex, age, income, education level and social class by occupation were collected online in the United Kingdom (UK; *N* = 1061) as part of the Food4Me survey. A detailed account of the survey methodology was published previously (Poínhos et al. 2014).

Respondents were asked to state their sex (male or female) and to indicate to which of four age groups (18 to 29; 30 to 39; 40 to 54; 55 to 65) they belonged. Education level was computed using the International Standard Classification of Education (ISCED) system and then classified into three groups (level 0 to 2 = low; level 3 to 4 = middle; level 5 to 6 = high). Annual household income was reported in pounds sterling on a 6-point scale (£0 to 11,000; £11,001 to 22,000; £22,001 to 33,000; £33,001 to 55,000; £55,001 to 88,000; > £88,000). A seventh group comprised those who chose not to declare their income. Social class by occupation was answered in response to an open-ended question ‘*please state your occupation*’. Occupation was then computed into nine groups using the Standard Occupational Classification (SOC) Hierarchy (Office for National Statistics – ONS) (higher managerial; lower managerial; intermediate, small employers; lower supervisory; semi-routine; routine; unemployed; and in education). A further category ‘unclassified’ contained cases where occupation had not been supplied.

The attitude scale comprised the four general attitude items from Crites et al. (1994). Each item was measured on a 5-point semantic differential scale in response to ‘personalised nutrition is’: 1. very valuable – very worthless; 2. very pleasant – very unpleasant; 3. very interesting – very boring; and 4. very good – very bad. The attitude scale showed good internal consistency (α = .87). The question on intention to adopt personalised nutrition comprised three items (Ajzen 1991). The items were I intend to adopt personalised nutrition; I would consider adopting personalised nutrition; I am definitely going to adopt personalised nutrition. Each item was assessed on a 5-point Likert scale ranging from 1 = completely disagree to 5 = completely agree. The scale showed good internal consistency (α = .91).

Between-groups multivariate analysis of variance (MANOVA) with the Tukey honestly significant difference (HSD) correction for multiple comparisons applied, was used to determine differences in attitude and intention to adopt personalised nutrition between sex, age (4 levels), education level (3 levels), income (7 levels) and occupational social class (10 levels). Data analyses were conducted using (IBM SPSS for Windows version 26.0). The *p* < 0.05 level was taken as significant.

## Results

Volunteers (*N* = 1061) were quota-sampled to be representative of the UK in terms of age (18–29 years = 23%; 30–39 years = 19.4%; 40–54 years = 36%; 55–65 years = 21.6%), sex (49% female) and education level (low = 49%; middle = 15.4%; high = 35.6%). Modal income was £11,001–22,000). Occupation was spread among the sample (Table 1).

**Table 1:** Sex, age, education level, income group, occupation and attitudes toward and intention to adopt personalised nutrition: results of multivariate analysis of variance (MANOVA) with Tukey HSD correction applied.

	n (%)	Attitude				Intention			
		*F (df)*	*EMM (SE)*	*p*	*Partial eta*_*2*_	*F (df)*	*EMM (SE)*	*p*	*Partial eta*_*2*_
OVERALL SAMPLE	1061 (100)		3.44 (0.03)				2.92 (0.04)		
Sex		12.569 (11039)		< 0.001	0.012	9.076 (11039)		0.003	0.009
Male	541 (51.0)		3.36 (0.04) [a]				2.84 (0.05) [a]		
Female	520 (49.0)		3.52 (0.04) [b]				3.00 (0.05) [b]		
Age group		10.433 (31039)		< 0.001	0.029	11.790 (31039)		< 0.001	0.033
18 to 29 years	244 (23.0)		3.63 (0.05) [a]				3.18 (0.06) [a]		
30 to 39 years	206 (19.4)		3.46 (0.05) [a,b]				2.94 (0.07) [b]		
40 to 54 years	382 (36.0)		3.45 (0.04) [b]				2.90 (0.05) [b]		
55 to 65 years	229 (21.6)		3.23 (0.05) [c]				2.65 (0.07) [c]		
Education level		0.308 (21039)		0.735	0.001	0.938 (21039)		0.392	0.002
Low	520 (49.0)		3.45 (0.04)				2.97 (0.05)		
Middle	163 (15.4)		3.41 (0.06)				2.86 (0.07)		
High	378 (35.6)		3.46 (0.04)				2.93 (0.05)		
Household annual income		0.647 (61039)		0.692	0.004	0.404 (61039)		0.877	0.002
£0 to £11,000	176 (16.6)		3.49 (0.06)				2.91 (0.08)		
£11,001 to £22,000	241 (22.7)		3.46 (0.05)				2.92 (0.06)		
£22,001 to £33,000	216 (20.4)		3.48 (0.05)				2.98 (0.06)		
£33,001 to £55,000	225 (21.2)		3.48 (0.05)				2.97 (0.06)		
£55,001 to £88,000	94 (8.9)		3.51 (0.08)				2.99 (0.09)		
More than £88,000	29 (2.7)		3.27 (0.13)				2.83 (0.16)		
Do not know/Will not say	80 (7.5)		3.39 (0.08)				2.85 (0.10)		
Occupation		0.923 (91039)		0.504	0.008	0.869 (91039)		0.553	0.007
Higher managerial, administrative, professional	44 (4.1)		3.65 (0.11)				3.04 (0.14)		
Lower managerial, administrative, professional	127 (12.0)		3.50 (0.07)				2.99 (0.08)		
Intermediate	86 (8.1)		3.41 (0.08)				2.96 (0.10)		
Small employers and self-employed	124 (11.7)		3.42 (0.07)				2.86 (0.08)		
Lower supervisory and technical	55 (5.2)		3.44 (0.10)				2.97 (0.12)		
Semi-routine	125 (11.8)		3.43 (0.07)				2.85 (0.08)		
Routine	121 (11.4)		3.34 (0.07)				2.81 (0.09)		
Long-term unemployed	216 (20.4)		3.36 (0.06)				2.81 (0.07)		
In education	58 (5.5)		3.44 (0.10)				3.05 (0.12)		
Cannot categorise	105 (9.9)		3.41 (0.08)				2.85 (0.10)		

EMM = estimated marginal means

Letters within square brackets indicate the homogeneous subsets (Tukey HSD). Values with the same character are not significantly different

Attitude: R2 = 0.060; adjusted R2 = 0.041; F(21,1039) = 3.154; p < 0.001

Intention: R2 = 0.062; adjusted R2 = 0.043; F(21,1039) = 3.278; *p* < 0.001

Between-groups MANOVA indicated that females had significantly more positive attitudes to personalised nutrition than males (*p <* 0.001; η_p_^2^ = 0.012) and were significantly more likely than males to intend to adopt personalised nutrition (*p* = 0.003; η _p_^2^ = 0.009) (Table 1).

There was a significant effect of age on attitudes towards (*p* < 0.001; η_p_^2^ = 0.029) and intention to adopt (*p* < 0.001; η _p_^2^ = 0.033) personalised nutrition. Post hoc tests indicated that younger participants had more positive attitudes towards personalised nutrition and were more likely to adopt it (Table 1).

No significant effect of education level was observed on attitudes toward (*p* = 0.735) or intention to adopt (*p* = 0.392) personalised nutrition. Nor were there any significant effects of income group on attitudes toward (*p* = 0.692) or intention to adopt (*p* = 0.877) personalised nutrition. There were no significant differences between occupation groups in either attitude (*p* = 0.504 or intention (*p* = 0.553) to adopt personalised nutrition (Table 1).

## Discussion

This analysis sought to identify socioeconomic differences in attitudes and intention to adopt personalised nutrition. The results imply that males may be less receptive to personalised nutrition and less likely to take it up in the future. The finding that males held less favourable attitudes and were less likely than females to intend to adopt personalised nutrition agrees with previous survey research on healthy eating (Hiller et al. 2017) and on personalised nutrition (Szakaly et al. 2021; 2016; Stewart-Knox et al. 2009). This, along with results from the wider Food4Me survey (Fischer et al. 2016) indicating that males were less willing to pay for personalised nutrition than females, implies that personalised, nutritional genomic intervention should target men and women differently (Corella et al. 2019).

Attitudes and intention to adopt personalised nutrition were found to vary by age. Attitudes were more favourable among younger individuals than among those who were older. People in the youngest age group (18–29 years) were also more likely than any of the older age groups to intend to adopt personalised nutrition in the future. The positive attitude of younger people is particularly promising, since previous research has indicated that younger people eat less healthy diets than those who are older (Kang et al. 2019; Roberts et al. 2018; Martinez-Lacoba et al. 2018; Ashton et al. 2017; van Dillen et al. 2008) and encounter greater barriers in achieving a healthy diet (Adams et al. 2019). A possible reason that younger people are more favourable toward personalised nutrition, therefore, could be that it is perceived to hold potential to overcome barriers to healthy eating. The effect sizes were appropriate for age differences in attitude or intention, which implies we can have confidence in these findings.

Attitudes toward personalised nutrition and intention to adopt it did not differ between education level, household income or occupation grouping in this UK population sample. This was unexpected given previous research to suggest that dietary quality is related to education level (Kang et al. 2019; Tan et al. 2017) and income (Roberts et al. 2018; Tan et al. 2017). These null findings are also contrary to those of a previous survey conducted in Hungary indicating that attitudes toward personalised nutrition were more favourable among those with a higher education level (Szakaly et al. 2016).

### What this study adds

This study enhances understanding of socioeconomic factors and receptiveness to personalised dietary health technology. This analysis appears to be among the first to have considered sex and age, education level, income, occupation with attitudes toward and intention to adopt personalised nutrition. That the sample was of sufficient size and representative in sex and age with a good spread across income and occupation, instills confidence in these findings.

### Limitations of this study

The study is not without certain limitations. Collecting data online may have biased the sample toward those more aware of digitally assisted health technologies. Inaccuracies inherent in self-report could have further biased responses toward what is perceived to be socially acceptable. Owing to limitations in the ability to categorise occupation manually in large samples, these data were collected within only one country, and this limits the generalisability of the results. There may also be inaccuracies associated with the UK SOC system used to classify occupation which may have affected the results.

Data were collected prior to the COVID-19 pandemic, and although they provide a baseline of pre-COVID attitudes and intention, lasting changes in attitudes and intention to adopt personalised nutrition may have occurred since the pandemic began. That those in less favourable socioeconomic circumstances are most severely affected by COVID-19 (Baena-Diez et al. 2021; Quan et al. 2021; Raisi-Estabragh et al. 2020) and experience the greatest food insecurity (Power et al. 2020), points to the increasing need for personalised nutrition provided as part of mainstream health services. The pandemic is also likely to have accentuated and accelerated societal need for technological solutions to dietary health promotion by necessitating social distance and increasing peoples’ awareness of dietary health. Given many members of the public are already accessing personalised health technologies through the commercial sector, any delay in widening access could exacerbate health-related inequality.

## Conclusion

This analysis suggests that males may be less likely than females to adopt personalised nutrition. Further in-depth enquiry is required to better understand sex differences in attitudes and intended adoption of dietary health technologies. That younger people held more positive attitudes and were more likely to intend to adopt personalised nutrition, bodes well for a future in which tech-enabled, personalised healthy eating services can be rolled out to all societal sectors as part of mainstream dietary health promotion. Harnessing technologies for individualised dietary health promotion and widening access to personalised nutrition to all sections of UK society could serve to narrow health inequalities, including those exacerbated by the COVID-19 pandemic.

## Data Availability

Materials and data set are available from the corresponding author upon request.
